# Multimodal Assessment of the Pulse Rate Variability Analysis Module of a Photoplethysmography-Based Telemedicine System

**DOI:** 10.3390/s21165544

**Published:** 2021-08-18

**Authors:** Flóra Antali, Dániel Kulin, Konrád István Lucz, Balázs Szabó, László Szűcs, Sándor Kulin, Zsuzsanna Miklós

**Affiliations:** 1 Institute of Translational Medicine, Semmelweis University, 1094 Budapest, Hungary; kulin.daniel@med.semmelweis-univ.hu; 2 E-Med4All Europe Ltd., 1036 Budapest, Hungary; luczkonrad@gmail.com (K.I.L.); medessence@gmail.com (B.S.); szucs.laszlo@scn4all.com (L.S.); dr.kulin.sandor@pregnascan.eu (S.K.); 3 Antal Bejczy Center for Intelligent Robotics, Óbuda University, 1034 Budapest, Hungary

**Keywords:** pulse rate variability, pulse wave analysis, photoplethysmography, telemedicine

## Abstract

Alterations of heart rate variability (HRV) are associated with various (patho)physiological conditions; therefore, HRV analysis has the potential to become a useful diagnostic module of wearable/telemedical devices to support remote cardiovascular/autonomic monitoring. Continuous pulse recordings obtained by photoplethysmography (PPG) can yield pulse rate variability (PRV) indices similar to HRV parameters; however, it is debated whether PRV/HRV parameters are interchangeable. In this study, we assessed the PRV analysis module of a digital arterial PPG-based telemedical system (SCN4ALL). We used Bland–Altman analysis to validate the SCN4ALL PRV algorithm to Kubios Premium software and to determine the agreements between PRV/HRV results calculated from 2-min long PPG and ECG captures recorded simultaneously in healthy individuals (n = 33) at rest and during the cold pressor test, and in diabetic patients (n = 12) at rest. We found an ideal agreement between SCN4ALL and Kubios outputs (bias < 2%). PRV and HRV parameters showed good agreements for interbeat intervals, SDNN, and RMSSD time-domain variables, for total spectral and low-frequency power (LF) frequency-domain variables, and for non-linear parameters in healthy subjects at rest and during cold pressor challenge. In diabetics, good agreements were observed for SDNN, LF, and SD2; and moderate agreement was observed for total power. In conclusion, the SCN4ALL PRV analysis module is a good alternative for HRV analysis for numerous conventional HRV parameters.

## 1. Introduction

The time duration between heart beats (interbeat intervals, IBIs) continuously changes, even at rest. These alterations are referred to as heart rate variability (HRV) and are brought about by various oscillating regulatory mechanisms that directly or indirectly affect heart rate (HR). These processes dominantly act by modifying the balance of sympathetic and parasympathetic effects on the heart; however, HR fluctuations due to other regulatory mechanisms (chemical, hormonal, and hemodynamic factors) also participate [1,2,3,4]. Control mechanisms contributing to HRV are diverse (e.g., respiratory rhythm, oscillations of baroreceptor activity, thermoregulation, etc.) and operate at different timescales [2,3,5,6]. In general, fluctuations of parasympathetic activity occur at higher frequencies, whereas those of sympathetic activity and hormonal effects at lower frequencies [6].

HRV analysis provides indices that characterize the variability of the IBIs (time-domain parameters) [2] and also that reflect the contribution of control mechanisms oscillating at different frequencies to this variability (frequency-domain parameters). In addition, so-called non-linear parameters that characterize the unpredictability of HR are also derived. HRV analysis is performed by analyzing normal-to-normal (i.e., non-arrhythmic) IBIs of sequential heartbeats acquired from continuous ECG recordings of various lengths (from 2 min to 24 h) [1,5,7]. In general, healthy people tend to have higher HRV values, which reflect the flexibility of regulatory systems to respond to different cardiovascular and homeostatic challenges [8,9,10,11,12,13], whereas depressed HRV has been associated with a wide variety of diseases and pathophysiological disorders. Moreover, alterations of certain HRV indices have been proposed to be applicable for assessment of prognosis in post-infarction patients and in patients with congestive heart failure [14,15,16,17,18,19,20,21,22,23,24,25,26].

These observations indicate that HRV analysis has a promising potential to evolve to a useful medical tool to monitor cardiovascular status. Since physiological fluctuations of autonomic functions make HRV parameters highly variable even within the same individual, HRV evaluation offers the most benefit if regular measurements are available. This can be easily accomplished by using telemedical and wearable monitoring systems equipped with HRV analysis modules. Nowadays, photoplethysmography (PPG)-based devices to monitor heart rate and oxygen saturation are very common both in clinical practice and everyday activities. PPG is a technique that detects blood volume changes in the tissues with an optical method. The PPG signal is an invaluable source of information of cardiovascular and autonomic functions. Among others, continuous PPG recordings obviously offer the opportunity to determine IBIs from which pulse rate variability (PRV) indices similar to HRV indices can be derived. However, it is debated whether PPG-based PRV indices can be interpreted similarly to HRV parameters, since IBIs are defined as RR intervals from ECG, and as pulse durations from PPG are not obviously identical. RR intervals signify the duration of the electrical cardiac cycle, which may slightly differ from PPG pulse durations (most often defined as peak-to-peak intervals of PPG pulse waves), since the timing of peripheral pulse peaks is influenced by several additional factors including the dynamics of ventricular ejection, elasticity of large arteries, peripheral resistance, and the propagation velocity of the pulse wave [27,28,29]. Moreover, this implies that PRV parameters may bear additional information about cardiovascular functioning, which is not available in HRV indices. Disparities between HRV and PRV have already been studied by several researchers, and various HRV and PRV indices have been reported to highly correlate in healthy individuals [30,31,32,33,34,35,36,37,38,39]. However, most of these studies are restricted to selected HRV parameters and resting healthy conditions. Comprehensive investigations covering numerous HRV indices [27,31,34] (including time-domain, frequency-domain, and non-linear parameters in the same study) and studies focusing on agreements between HRV and PRV parameters under autonomic challenge [39] and in diseased conditions are scarce in the literature.

Our research group has recently introduced a telemedical system (SCN4ALL) that is designed for the remote monitoring of cardiovascular patients and is based on the photoplethysmographic (PPG) detection and analysis of the digital arterial pulse wave [40]. The system analyzes continuous 2-min long PPG recordings, which are used to evaluate morphological pulse characteristics [40]. In order to offer the most benefit for our users, we have also elaborated an automated algorithm for PRV computation and equipped the telemedical system with a PRV analysis module. The ultimate aim of this study is the comprehensive assessment of the performance of the SCN4ALL pulse rate variability analysis module.

First, we aimed to assess the agreements between the most widespread conventional HRV and PRV indices computed from ECG and PPG captures, respectively. For this purpose, we simultaneously recorded ECG and PPG on healthy individuals at rest and also under cold pressor challenge, when the autonomic balance was disrupted. We calculated IBIs and 17 HRV parameters from both captures using a clinically validated and widely accepted algorithm (Kubios HRV Premium) [41] and then compared the results with Bland–Altman analysis. The agreements of HRV and PRV parameters were also investigated in diabetic patients in order to assess if the interchangeability observed in healthy individuals also holds for diseased conditions.

Automated algorithms used for HRV analysis use slightly different mathematical approaches for the power spectral and non-linear analysis of HRV. In this study, we also aimed to validate our proprietary SCN4ALL algorithm to a clinically accepted algorithm in order to show its reliability before introduction to clinical research and practice. For this purpose, we performed PRV analysis on 2-min long PPG recordings both with the Kubios HRV Premium [41] and the SCN4ALL algorithms and compared the results with Bland–Altman analysis.

## 2. Materials and Methods

### 2.1. Subjects

A total of 33 informed and consenting healthy (M/F: 14/19, age between 19 and 55, mean ± SD: 32.1 ± 9.7 years) and 12 type 2 diabetic (M/F:5/7, age between 43–79, mean ± SD: 61.1 ± 12.8 years) subjects participated in this study. None of the healthy volunteers had a history of cardiovascular disease, or cardiovascular medication, and none of them reported any symptoms that may affect autonomic balance (sleep deprivation, stress, headache, etc.). The participating diabetic patients had been treated for type 2 diabetes for more than one year. The study was approved by the Regional and Institutional Committee of Science and Research Ethics at Semmelweis University (approval number: 120/2018).

### 2.2. Measurements of HRV

#### 2.2.1. Signal Recording

**ECG.** Einthoven II lead ECG was recorded with the Biopac BSL MP45 data acquisition system (Biopac Systems Incl., Goleta, CA, USA). For ECG recording, disposable ECG electrodes were attached to the right shoulder, left lower abdomen, and right lower abdomen, and then connected to the negative, positive, and ground wires of a Biopac SS2LB electrode lead set, respectively. The signal was amplified by a Biopac MP45 data acquisition unit, which was directly connected to a desktop computer. BSL 3.7.7 software was used to capture ECG for 2 min at a sample rate of 1000 Hz. ECG recordings were saved as *.acq* files and were used to identify RR intervals by the Kubios HRV Premium analysis software (Kubios Ltd., Kuopio, Finland) [41]. RR intervals were used as IBIs (IBI-ECG) to calculate HRV parameters (for details, see data analysis).

**PPG.** For recording the PPG signal, a finger-clip transmission pulse oximeter (Berry Pulse Oximeter, Shanghai Berry Electronic Tech Co., Ltd., Shanghai, China) was attached to the left index finger. Pulse wave detection and analysis were performed by the SCN4ALL telemedicine system (E-Med4All Europe Ltd., Budapest, Hungary). In the system, the pulse oximeter communicates via Bluetooth connection with a mobile application, which initiates and terminates data acquisition and transmits the recording to a cloud-based automated algorithm, which has been developed by our research group. First, the signals sampled at a frequency of 200 Hz are upsampled to 1000 Hz; then, the algorithm identifies the pulse cycles and peak-to-peak intervals as IBIs (IBI-PPG). Time series of IBI-PPG were used to calculate PRV parameters (for details, see data analysis). Data captured by the SCN4ALL system is stored on a cloud-based server equipped with safe data protection, which conforms to the applicable regulations ((EU)2016/679) [40].

#### 2.2.2. Protocol

We performed the measurements on 33 healthy and 12 diabetic participants under the following conditions: measurement took place in a quiet room at room temperature, in a sitting, resting position, with hands held quietly on a table. The pulse oximeter was placed on the left index finger, and the ECG electrodes were attached as described above. After mounting the devices, participants were instructed to minimize movements. Measurements were initiated after 10 min of rest. First, we measured the blood pressure of the participants with an Omron M3 Intellisense arm-cuff blood pressure meter 3 times, with 2-min intervals between the measurements (OMRON Corporation, Kyoto, Japan). Afterwards, ECG and PPG signals were simultaneously recorded for 2 min using Biopac 3.7.7 software and SCN4ALL application, respectively.

After completion of the resting examination, the healthy volunteers remained seated, and we repeated the measurements in these subjects also during a cold pressor cardiovascular challenge, which was applied to disturb the resting autonomic balance. First, we measured the participants’ blood pressures. Then, the right hand was immersed in a bowl of cold water (+3–5 °C). ECG and PPG recordings were initiated simultaneously with the cold pressor challenge and lasted for 2 min. Blood pressure measurements were repeated immediately after the termination of the 2-min recording period.

### 2.3. Data Analysis

In the analysis, only those recordings were evaluated that consisted of normal-to-normal IBIs. For this reason, results of 3 healthy subjects were completely excluded from the study, because in 2 cases, previously unknown arrhythmia was seen on the ECG, and in 1 case, the PPG was contaminated with motion artefacts. Four additional healthy subjects were excluded from the ‘cold pressor test’ study either for not tolerating the challenge or for producing numerous PPG artefacts. Therefore, in the healthy group, results of control measurements are presented for 30 (M/F: 14/16, age range 19–55, mean ± SD: 33 ± 9.7 years), and those of the cold pressor study for 26 subjects (M/F: 11/15, age range 19–55, mean ± SD: 33 ± 9.9 years).

We calculated HRV/PRV parameters from the two detection modalities (ECG and PPG) by three methods:

1. ECG recordings (.*acq* files captured by the Biopac system) were opened in Kubios HRV Premium software (ver. 3.3.1), which identified RR intervals (IBI-ECG) and then computed HRV parameters. As only non-arrhythmic recordings were used, the calculations were made using no artefact correction and with unfiltered settings. As a result, HRV-ECG values were generated.
2. We saved peak-to-peak intervals calculated by the SCN4ALL algorithm from each PPG recording (IBI-PPG) as *.csv* files. The PRV analysis of the IBI-PPG datasets were executed with Kubios HRV Premium, with the same settings as in Point 1. As a result, PRV-Kubios values were created.
3. The automatic algorithm of the SCN4ALL system was also used to calculate PRV parameters from IBI-PPG data to produce PRV-SCN4ALL values. The functions of the algorithm were programmed in Matlab. The algorithm uses the statistical approaches recommended by the ‘Task Force of the European Society of Cardiology and the North American Society of Pacing Electrophysiology’ [5] to determine time-domain parameters. For frequency-domain analysis, a power spectrum density estimate was calculated by the algorithm using a Fast Fourier Transform (FFT)-based Welch’s periodogram method. After obtaining the FFT spectrum, absolute power values for each frequency band were calculated by simply integrating the spectrum within the band limits. To compute non-linear PRV parameters, detrended fluctuation analysis was performed according to the work of C.G Peng et al. [42]. SCN4ALL also displays a Poincaré plot with SD1 and SD2 parameters. Poincaré plot is a graph of IBI(n) on the *x*-axis versus IBI(n + 1) on the *y*-axis [43,44]. SD1 is the standard deviation of the distance of the points from the “x = y” axis and reflects short-term changes, whereas SD2 is the standard deviation of the distance of the points from the “x = −y + 2xIBI(mean)” axis [44,45]. SD1 and SD2 determine the length and width of a fitted ellipsis, respectively, the center of which is at the coordinate of (IBI(mean);IBI(mean)). In fact, SD1 and SD2 can be mathematically derived from time-domain indices; therefore, we calculated SD1 and SD2 as follows [44,46,47,48]:

$$\text{SD}1\;=\;\text{rMSSD}\times \frac{1}{\sqrt{2}}$$

$$\text{SD}2\;=\;\sqrt{2\times {\text{SDNN}}^{2}-\text{SD}1^{2}}$$

Comparison of the HRV and PRV results derived according to points 1 and 2 describes the agreement between the ECG and PPG methodologies (performed for healthy individuals at rest and during cold pressor test and for diabetic subjects at rest). In contrast, comparison of the PRV results between points 2 and 3 provides information about the performance of the SCN4ALL PRV analysis engine compared to the widely used and clinically accepted Kubios HRV Premium analysis [41]. The analysis was performed for a wide range of HRV/PRV parameters, which are listed in Table 1.

### 2.4. Bland–Altman Analysis

The agreements between HRV/PRV parameter values (HRV-ECG vs. PRV-Kubios and PRV-Kubios vs. PRV-SCN4ALL) were assessed by Bland–Altman analysis [49,50]. The differences of measurements were plotted against the means of the measurements. Bias was defined as mean difference and is presented with 95% confidence intervals (C.I.). To calculate percentage bias, bias is expressed as the percentage of the mean of the measurements. Limits of agreement were calculated as bias ± 1.96 standard deviation. The analysis was performed with MedCalc Statistical Software v.19.6.4 (MedCalc Software, Ostend, Belgium).

## 3. Results

### 3.1. Agreements between ECG-Based HRV and PPG-Based PRV Parameters

**Figure 1:**
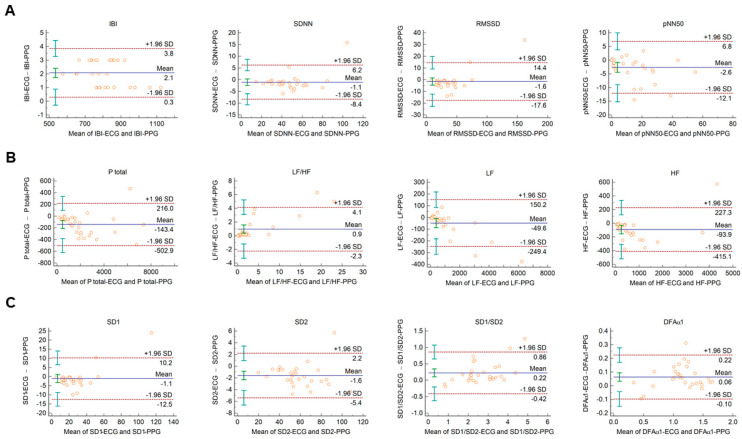
Bland–Altman plots of HRV/PRV parameters computed by the Kubios Premium algorithm from 2-min long ECG (indicated as ‘parameter name-ECG’) and PPG (indicated as ‘parameter name-PPG’) recordings captured under resting conditions. (**A**) Time-domain parameters: IBI (interbeat interval), SDNN (the standard deviation of IBIs), RMSSD (the square root of the mean squared differences of successive IBIs), pNN50 (the proportion of differences of successive IBIs exceeding 50 ms). (**B**) Frequency-domain parameters: Ptotal (total spectral power), LF/HF (ratio of low frequency to high frequency), LF (absolute power of the low-frequency band (0.04–0.15 Hz)), HF (absolute power of the high-frequency band (0.15–0.4 Hz)). (**C**) Non-linear parameters: SD1 (Poincaré plot standard deviation perpendicular to the line of identity), SD2 (Poincaré plot standard deviation along the line of identity), SD1/SD2 (ratio of SD1-to-SD2), DFAα1 (short term fluctuation slope obtained by detrended fluctuation analysis). Bias is calculated as the mean of differences (indicated as ‘Mean’—blue solid line) and is presented with 95% confidence intervals (green) and +/− 1.96 standard deviations (SD) and their confidence intervals.

The Bland–Altman plots used for the analysis of agreement between PRV and HRV parameters derived from 2-min long PPG and ECG recordings, respectively, are shown in Figure 1. In this setting, conventional HRV indices were calculated by the algorithm of the Kubios HRV Premium software. The values of variables referring to IBI duration (IBI, mean HR, minimum HR, and maximum HR) are apparently identical in PPG and ECG based calculations (Figure 1A, and Supplementary Figure S1A). Among time-domain parameters, SDNN and RMSSD showed good agreement. The percentage biases were −3.2% (95% C.I.: −5.2; −1.2) and −9.5% (95% C.I.: −14.3; −4.6), respectively, indicating that the values calculated from PPG recordings are slightly higher. However, in the case of pNN50, the bias was −27.3% (95% C.I.: −53.2; −1.4) (Figure 1A). Among frequency-domain parameters, good agreement was observed for total and low-frequency spectral power (percentage bias −8.2% (95% C.I.: −10.6; −5.8) for total power (Ptotal); and −2.7% (95% C.I.: −4.9; −0.5) for LF) (Figure 1B and Supplementary Figure S1B)). However, the agreement for high-frequency power was weaker (percentage bias −26.5% (95% C.I.: −35.6; −17.5) (Supplementary Figure S1B)), with significant overestimation of the parameter by the PPG based calculation. The calculated non-linear parameters (DFAα1, SD1, SD2, and SD1/SD2) each showed good agreement (Figure 1C).

**Figure 2:**
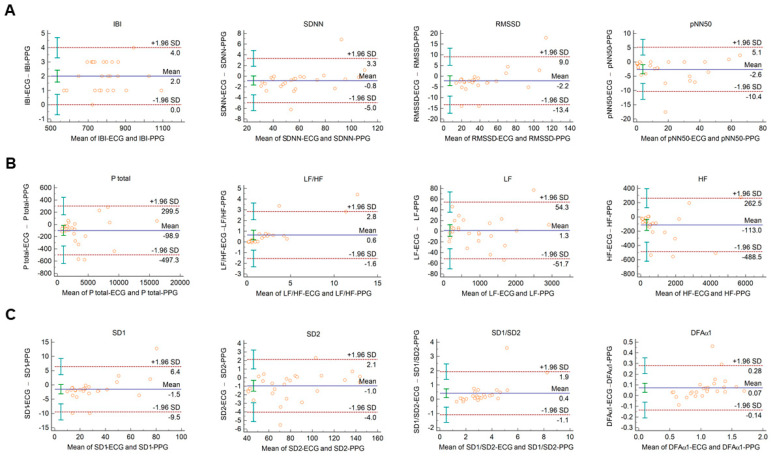
Bland–Altman plots of HRV/PRV parameters computed by the Kubios Premium algorithm from 2-min long ECG (indicated as ‘parameter name-ECG’) and PPG (indicated as ‘parameter name-PPG’) recordings obtained during cold pressor test. (**A**) Time-domain parameters: IBI (interbeat interval), SDNN (the standard deviation of IBIs), RMSSD (the square root of the mean squared differences of successive IBIs), pNN50 (the proportion of differences of successive IBIs exceeding 50 ms). (**B**) Frequency-domain parameters: Ptotal (total spectral power), LF/HF (ratio of low frequency to high frequency), LF (absolute power of the low-frequency band (0.04–0.15 Hz)), HF (absolute power of the high-frequency band (0.15–0.4 Hz)). (**C**) Non-linear parameters: SD1 (Poincaré plot standard deviation perpendicular to the line of identity), SD2 (Poincaré plot standard deviation along the line of identity), SD1/SD2 (ratio of SD1-to-SD2), DFAα1 (short-term fluctuation slope obtained by detrended fluctuation analysis). Bias is calculated as the mean of differences (indicated as ‘Mean’—blue solid line) and is presented with 95% confidence intervals (green) and +/− 1.96 standard deviations (SD) and their confidence intervals.

When Bland–Altman analysis was performed on HRV vs. PRV parameters calculated from 2-min ECG and PPG recordings obtained from healthy individuals during cold pressor test, similar tendencies could be observed with similar IBI durations, and with good, clinically acceptable agreements for SDNN, RMSSD, total power, and LF; and also, for non-linear parameters (Figure 2 and Supplementary Figure S2.).

**Figure 3:**
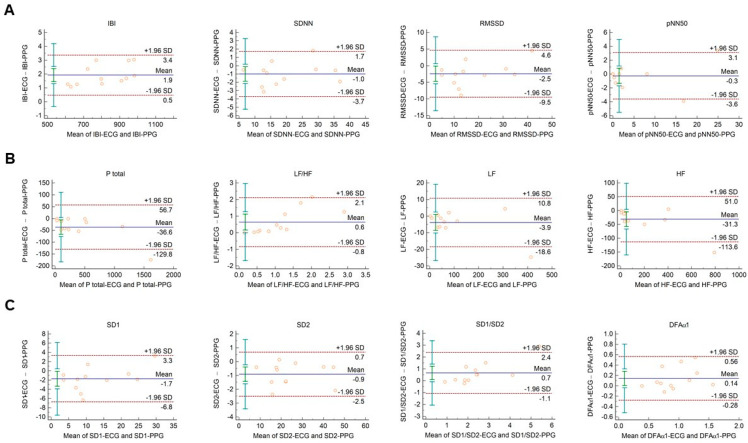
Bland–Altman plots of HRV/PRV parameters computed by the Kubios Premium algorithm from 2-min long ECG (indicated as ‘parameter name-ECG’) and PPG (indicated as ‘parameter name-PPG’) recordings obtained from diabetic patients under resting conditions. (**A**) Time-domain parameters: IBI (interbeat interval), SDNN (the standard deviation of IBIs), RMSSD (the square root of the mean squared differences of successive IBIs), pNN50 (the proportion of differences of successive IBIs exceeding 50 ms). (**B**) Frequency-domain parameters: Ptotal (total spectral power), LF/HF (ratio of low frequency to high frequency), LF (absolute power of the low-frequency band (0.04–0.15 Hz)), HF (absolute power of the high-frequency band (0.15–0.4 Hz)). (**C**) Non-linear parameters: SD1 (Poincaré plot standard deviation perpendicular to the line of identity), SD2 (Poincaré plot standard deviation along the line of identity), SD1/SD2 (ratio of SD1-to-SD2), DFAα1 (short-term fluctuation slope obtained by detrended fluctuation analysis). Bias is calculated as the mean of differences (indicated as ‘Mean’—blue solid line) and is presented with 95% confidence intervals (green) and +/− 1.96 standard deviations (SD) and their confidence intervals.

In diabetic individuals, the Bland–Altman analysis showed good agreements between HRV and PRV values for IBI durations (Figure 3A), SDNN (Figure 3A), LF power (Figure 3B), and SD2 variables (Figure 3C) (percentage bias < 10% for each parameter). Slightly weaker, moderate agreements were observed for total power (Figure 3B; percentage bias −14.2% (95% C.I.: −23.3; −5.1)); and for DFAα1 non-linear parameter (Figure 3C; percentage bias 13.8% (95% C.I.: 0.0; 27.6)). However, in case of RMSSD and pNN50 time-domain variables (Figure 3A); HF and relative (HFnu, LFnu, LF/HF) frequency-domain indices (Figure 3B and Supplementary Figure S3); and SD1 and SD1/SD2 non-linear parameters (Figure 3C), the agreements were found to be insufficient (percentage bias > 20%).

### 3.2. Agreements between PRV Calculations of the SCN4ALL and Kubios HRV Premium Algorithms

**Figure 4:**
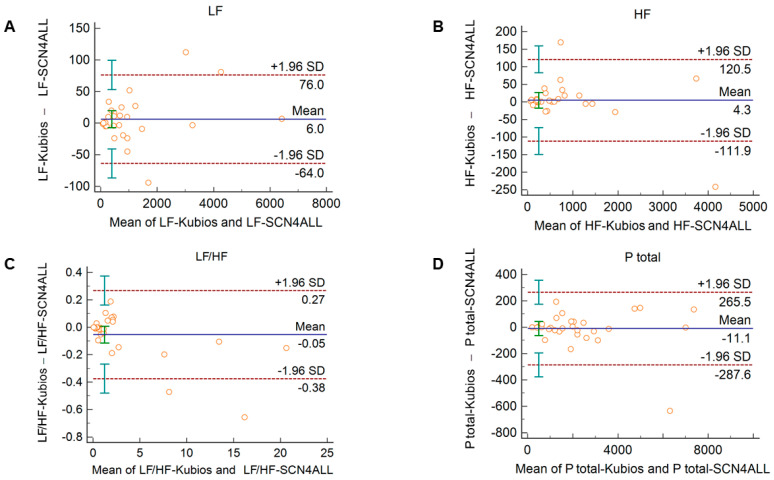
Bland–Altman plots of frequency-domain HRV/PRV parameters calculated by the SCN4ALL (indicated as ‘parameter name-SCN4ALL’) and the Kubios Premium HRV (indicated as ‘parameter name-Kubios’) algorithms from 2-min long PPG recordings captured under resting conditions. (**A**) Ptotal (total spectral power), (**B**) LF/HF (ratio of low frequency to high frequency), (**C**) LF (absolute power of the low-frequency band (0.04–0.15 Hz)), (**D**) HF (absolute power of the high-frequency band (0.15–0.4 Hz)). Bias is calculated as the mean of differences (indicated as ‘Mean’—blue solid line) and is presented with 95% confidence intervals (green) and +/− 1.96 standard deviations (SD) and their confidence intervals.

Comparison of the PRV parameters calculated by the SCN4ALL and the Kubios HRV Premium algorithm from 2-min long PPG recordings showed perfect agreement in case of all PRV variables. For time-domain and non-linear variables (Supplementary Figure S3), the percentage biases were smaller than 0.5%. In case of frequency-domain variables, these values were below 2% and well within the clinically acceptable limits and with no significant difference between the outputs of the two algorithms (Figure 4).

**Figure 5:**
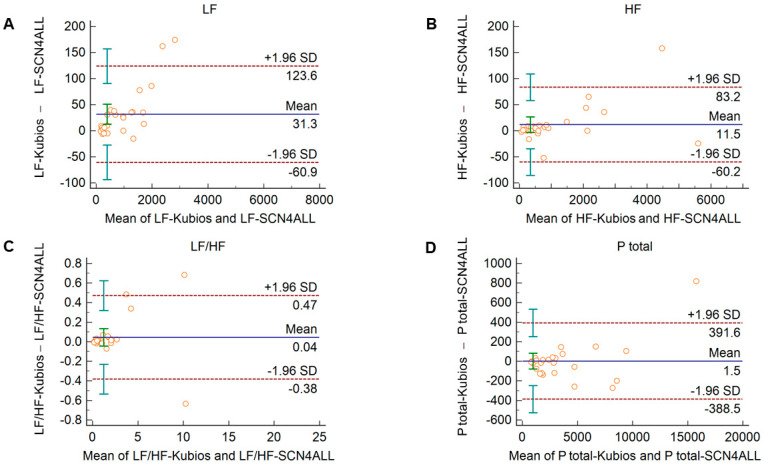
Bland–Altman plots of frequency-domain HRV/PRV parameters calculated by the SCN4ALL (indicated as ‘parameter name-SCN4ALL’) and the Kubios Premium HRV (indicated as ‘parameter name-Kubios’) algorithms from 2-min long PPG recordings obtained during cold pressor test. (**A**) Ptotal (total spectral power), (**B**) LF/HF (ratio of low frequency to high frequency), (**C**) LF (absolute power of the low-frequency band (0.04–0.15 Hz)), (**D**) HF (absolute power of the high-frequency band (0.15–0.4 Hz)) Bias is calculated as the mean of differences (indicated as ‘Mean’—blue solid line) and is presented with 95% confidence intervals (green) and +/− 1.96 standard deviations (SD) and their confidence intervals.

**Figure 6:**
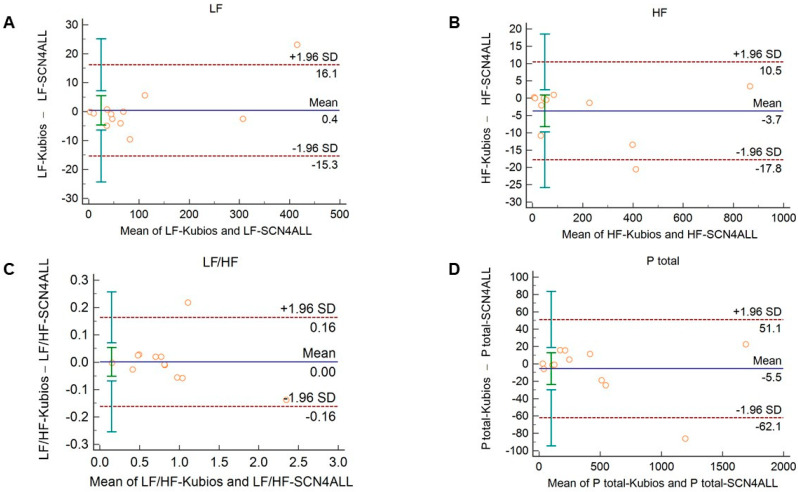
Bland–Altman plots of frequency-domain HRV/PRV parameters calculated by the SCN4ALL (indicated as ‘parameter name-SCN4ALL’) and the Kubios Premium HRV (indicated as ‘parameter name-Kubios’) algorithms from 2-min long PPG recordings obtained from diabetic patients under resting conditions. (**A**) Ptotal (total spectral power), (**B**) LF/HF (ratio of low frequency to high frequency), (**C**) LF (absolute power of the low-frequency band (0.04–0.15 Hz)), (**D**) HF (absolute power of the high-frequency band (0.15–0.4 Hz)). Bias is calculated as the mean of differences (indicated as ‘Mean’—blue solid line) and is presented with 95% confidence intervals (green) and +/− 1.96 standard deviations (SD) and their confidence intervals.

The agreement between the outputs of the algorithms remained unaltered when 2-min long recordings acquired in healthy subjects during cold pressor test (Figure 5 and Supplementary Figure S5) and in diabetic patients at rest (Figure 6 and Supplementary Figure S6) were used for analysis.

## 4. Discussion

In our study, using Bland–Altman plots, we have shown that PRV and HRV calculations (obtained from PPG and ECG recordings, respectively) are in good agreement for several conventional HRV/PRV parameters when the analysis is performed using short (2-min long) recordings. Apparently, there is no significant difference in mean interbeat intervals defined from PPG and ECG captures, and for several HRV/PRV parameters computed by the Kubios software, the limits of agreement are within 10% (i.e., SDNN and RMSSD among time-domain variables, total power and LF frequency-domain indices, and non-linear parameters). The agreement of HRV parameters obtained by the two methods prevailed even if the resting autonomic balance had been disrupted by a cardiovascular challenge (cold pressor test). In diabetic individuals, the good agreements between HRV and PRV indices were also valid for SDNN, LF, and SD2 indices, and moderate agreements could be detected between total spectral power and DFAα1 values. However, for parameters that are considered to be conventional markers of short-term HRV, weaker agreements were found. We have also shown that the outputs of the PRV algorithm of the SCN4ALL telemonitoring system are in perfect agreement with the values computed by Kubios HRV Premium when the analysis is performed on data derived from short (2-min long) PPG captures. Our study extends our scientific knowledge about the interchangeability of HRV and PRV analysis with relevant new pieces, as it is a comprehensive investigation covering a large number of HRV/PRV parameters and assessing their agreements not only in healthy individuals at rest but also under autonomic challenge and in diabetes.

Autonomic function has been in the focus of research for decades, and several non-invasive techniques have been proposed for its evaluation (ECG, PPG, electroencephalography, sudomotor function, etc.) [51,52,53]. Many of these may also be incorporated in remote monitoring systems, and experiences acquired in signal analysis of one method have often facilitated progression in the procession of other signals. Using the HRV analysis approach for PPG signals is a good example of this. However, in the literature, it is controversial whether HRV parameters calculated from time series of RR intervals obtained from ECG recordings and from pulse durations obtained from PPG signals or continuous non-invasive blood pressure monitoring (e.g., Finapress) can be used alternatively [54]. Nowadays, the number of wearable and telemedical devices that are equipped with either ECG or PPG detectors dynamically increases [55,56]. This may open new prospects for scientists and physicians to exploit the opportunities offered by HRV/PRV analysis in patient evaluation. However, most of our scientific knowledge on HRV alterations in different (patho)physiological conditions relies on ECG-based studies, mostly following a task force statement of the European Society of Cardiology and the North American Society of Pacing Electrophysiology [5]. Therefore, it is important to assess the agreement between HRV and PRV under different (physiological and pathological) conditions in order to confidently accept the PPG-based PRV-analysis as a reliable alternative to monitor HRV changes. So far, several studies have compared PRV to the gold standard of ECG-derived HRV [30,31,32,33,34,35,36,37,38,39]. Some publications found good agreements between PRV and HRV, especially in younger subjects and at rest [31,57], or during sleep [58], and mostly in time-domain parameters. However, some studies have found weaker agreements between HRV and PRV values for HRV indices, which are generally influenced by short-term regulatory fluctuations (RMSSD, pNN50, HF, LF/HF, SD1) [32,34,36,54,59,60,61]. Their results indicate that PRV overestimates HF but underestimates LF/HF ratio and LF percentage. However, it is notable that this is observed more often in continuous blood pressure monitoring studies (Finapress) than in PPG studies. There is sparse evidence of whether frequency-domain PRV variables behave similarly to HRV variables and have some value in diagnosing autonomic function [38,62]. In our study, we have shown that among time-domain variables, PPG-based and ECG-based SDNN and RMSSD values have good agreements (Figure 1A). Similar to previous studies, pNN50 was overestimated when PPG-based IBIs were used [27,32,34,36,54,59]. On the other hand, total spectral power and low-frequency power computed from PPG and ECG had similar values (Figure 1B). Interestingly, high-frequency power was significantly overestimated by the PPG-based analysis (Figure 1B). This is in agreement with some studies, in which similar observations were made for certain frequency-domain variables [32,34,36,39,54,60,61,63]. It has been speculated that the reason for this disparity in HF power and other indices reflecting short-term variability is that spontaneous breathing rate lying within the HF frequency band has a greater impact on PRV than on ECG-based HRV [54,59,62,64].

We also observed good agreements for non-linear parameters. The relevance of these parameters in HRV analysis is not completely established, and there is no consensus on the measurement duration which can yield clinically informative non-linear variables [65,66,67]. Moreover, some of these parameters are in a direct mathematical relationship with other parameters and bear the same information (e.g., SD1 and RMSSD). Anyway, our results show that PPG-based PRV analysis is a good alternative for HRV analysis in case of non-linear parameters, too.

Although several studies have shown correlations between PRV and HRV variables, these were observed at rest or during sleep. However, exercise, stress, or changing position were observed to diminish these agreements. The authors speculated that in physically active states, the disagreement is most probably due to motion artefacts [34,39]. On the other hand, the disparity between PRV and HRV variables can also be the consequence of the altered autonomic balance, which may affect pulse rate and heart rate differentially. In our study, we used the cold pressor cardiovascular challenge to disrupt the resting autonomic balance. This allowed examination of the effects of altered autonomic function without producing motion artefacts. Although not every subject had the same usual and expected cardiovascular response during the test, there was some disruption of the autonomic balance in every case (average increase in systolic pressure: 5.4 ± 7.7 mmHg, average increase in diastolic pressure: 3.7 ± 6.6 mmHg). The agreements in the PPG- and ECG-based analysis described at rest could also be observed during the cold pressor test (Figure 2, and Supplementary Figure S2), implying that PPG-based PRV analysis can be applicable also in conditions in which altered autonomic function has been described by HRV analysis. In our study, we chose a cold pressor test to modify autonomic balance, because this allowed us to avoid undesirable motion artefacts. However, this may have limitations, as in another study it has been shown that whole-body cold exposure has differential effects on HRV and PRV parameters, thereby modifying the agreements between them [27]. It was speculated that this can be most probably due to the unbalanced influence of cold exposure on central and peripheral sympathetic activity. In our study, cold exposure on one hand did not abolish the agreements of HRV and PRV parameters, presumably because its effects differ from those of whole-body cold exposure.

We have also conducted a pilot study to assess the agreements between HRV and PRV indices in type 2 diabetic patients in order to find out whether the agreements observed in healthy individuals are also valid in a diseased condition. Diabetes is characterized by reduced total and LF power, and also by the decrease of HRV parameters that signify mainly short-term variability (SDNN, RMSSD, pNN50, HF) [68,69,70,71]. These alterations are caused by the deleterious effects of the impaired glucose metabolism on autonomic nerves [70]. We found that for several relevant HRV parameters, such as SDNN, LF power, and SD2 parameters, good agreements can be detected between HRV and PRV derived values. Moreover, we observed moderate agreements (bias < 15%) in case of DFAα1 and total power. However, in case of those parameters that describe short-term variability (RMSSD, pNN50, SD1) though both HRV and PRV values tended to be lower in the diabetic group, the HRV-PRV agreements were weaker than those observed in healthy individuals. Our results suggest that several conventional HRV/PRV parameters can be used interchangeably not only in healthy but also in diabetic individuals; however, there are other parameters with non-negligible disparities. It does not necessarily imply that those parameters that show weaker agreements in our study are not worth evaluating. However, our findings highlight the relevance of larger-scale comparative HRV vs. PRV studies to verify whether diabetes or various other disease conditions are associated with typical alterations of these PRV variables. Data mining techniques to identify correlations between PRV patterns and different diseases could effectively improve our scientific knowledge in this field. As a result, we may identify the differences even in localized autonomic responses accounting for HRV and PRV disparities in order to establish sound diagnostic indications for HRV and PRV analyses.

HRV algorithms used for calculation of HRV variables may apply different mathematical approaches. This may limit the comparison of studies and the valid interpretation of the HRV variables and their alterations in different conditions. Therefore, we considered it to be relevant to validate our algorithm to a clinically widely accepted and frequently used HRV algorithm, the Kubios HRV Premium. In case of time-domain variables, we should expect perfect agreement between algorithms, since these parameters are calculated as statistical parameters describing IBI variability using formulae recommended by a task force statement [5]. However, for spectral analysis, two main different approaches can be used to separate HRV into frequency components, namely Fast Fourier Transformation (FFT) and autoregressive modeling [5]. For each approach, several slightly different functions can be applied. The SCN4ALL algorithm uses an FFT-based Welch’s periodogram method, which is similar to the one applied by the Kubios algorithm. For calculation of non-linear parameters, the SCN4ALL algorithm uses detrended fluctuation analysis according to the work of Peng et al. [42] and a Poincaré plot, which are characterized by SD1 and SD2 parameters defined in the “Methods” section above. Comparison of the SCN4ALL algorithm outputs to the Kubios outputs by Bland–Altman analysis showed perfect agreement between the methods when we analyzed 2-min long PPG-based IBI time series obtained from either healthy individuals at rest and during cold pressor cardiovascular challenge or from diabetic patients at rest. In case of those parameters where a simple mathematical formula is applied (time-domain variables, SD1 and SD2 non-linear variables), the negligible differences between the SCN4ALL and Kubios results are attributable to slightly different rounding schemes used by the algorithms.

Signal processing of telemedical systems may be prone to signal loss and uncertainty due to multistep signal transformation [72,73]. This can be interpreted as the uncertainty of the data used for classification. Effective classification of evidence requires the use of fuzzy classifiers [74,75]. Based on multiple studies [76,77,78], the fuzzy data application allows to increase the accuracy of the classification of uncertain data [79]. In the case of the PPG-based system used in our study, there are two possible steps where signal loss may occur. The first is the analog-to-digital conversion of the signal. In the case of heart rate variability, only the quantization error can play a role. The SCN4ALL telemedicine system operates at a sampling rate of 200 Hz, meaning that at a heart rate of 60 beats/minutes, it only creates a 0.5% error. This is clinically acceptable and does not affect the diagnostic value of the given system. The second step where some information loss can be expected is at the filtering of the digitized signal. However, it only affects the morphology of the PPG signal but not the timely relations of the fiducial timepoints. Therefore, filtering the signal does not affect the peak-to-peak distances of the pulse wave from which IBIs for PRV calculation are derived. Furthermore, in our previous article [40], we examined how artificial non-variable PPG signals generated by a simulator (both normal and simulated pathological signals) were processed by the system, and the repeatability was found to be perfect in case of most studied parameters [40]. Although this study focused on morphological parameters, we also investigated the reliability of IBI determination, and the error (expressed as coefficient of variation) was virtually zero. Our PRV analysis module uses only IBIs as detected signals for further computation, so we think that signal loss and uncertainty have a negligible effect on our analysis.

## 5. Conclusions

Our study showed that the HRV algorithm of the SCN4ALL system is as accurate as the widely used Kubios HRV Premium algorithm for PRV analysis of short (2-min long) time series of interbeat intervals obtained by PPG recordings. PRV analysis performed on PPG pulse signals is in good agreement with ECG-based analysis for numerous clinically relevant HRV parameters, including SDNN and RMSSD time-domain parameters, total and low-frequency spectral power frequency-domain variables, and non-linear parameters in healthy individuals at rest, and also under an autonomic challenge. Moreover, we identified several parameters (SDNN, total power, LF, SD2, and DFAα1) that showed moderate to good HRV-PRV agreements in diabetic patients. This indicates that these parameters can be reliably used for HRV-based evaluation of autonomic function in healthy and diabetic individuals regardless of whether ECG or PPG provides the time series of interbeat intervals. Other conventional PRV parameters computed from PPG recordings should be interpreted cautiously, keeping in mind that clinical evidence obtained on ECG-based HRV alterations in different disease conditions can be applied with limitations. Despite these limitations, we can claim that PPG-based PRV analysis of the SCN4ALL system is suitable for evaluation of PRV alterations, and to pursue research to establish the clinical relevance of PRV analysis in the follow-up of autonomic dysregulation in various diseases.

## Figures and Tables

**Table 1:** Heart rate variability parameters analyzed in the study.

**Time-Domain Parameters**	
Mean IBI	The mean normal-to-normal interbeat interval (IBI)
SDNN	The standard deviation (SD) of IBIs (NN: normal-to-normal IBI)
MHR	Mean heart rate
RMSSD	The square root of the mean squared differences of successive IBIs
pNN50	The proportion of differences of successive IBIs exceeding 50 ms (NN: normal-to-normal IBI)
MnHR	Minimum heart rate
MxHR	Maximum heart rate
**Frequency-Domain Parameters**	
LF power	Absolute power of the low-frequency (LF) band (0.04–0.15 Hz)
HF power	Absolute power of the high-frequency (HF) band (0.15–0.4 Hz)
LFnu	Relative power of the low-frequency (LF) band expressed in normalized units (nu)
HFnu	Relative power of the high-frequency (HF) band expressed in normalized units (nu)
Ptotal	Total spectral power (P)
LF/HF ratio	Ratio of low frequency (LF) to high frequency (HF)
**Non-Linear Parameters**	
SD1	Standard deviation (SD) 1 of the Poincaré plot representing the length of the ellipse fitted to the plot
SD2	Standard deviation (SD) 2 of the Poincaré plot representing the width of the ellipse fitted to the plot
SD1/SD2	The ratio of SD1 and SD2
DFAα1	Short term fluctuation slope (α1) obtained by detrended fluctuation analysis (DFA)

## Data Availability

The data that support the findings of this study are available from E-Med4All Europe Ltd., but restrictions apply to the availability of these data, which were used under license for the current study, and so are not publicly available. Data are however available from the authors upon reasonable request and with permission of E-Med4All Europe Ltd.

## Supplementary Materials

The following are available online at https://www.mdpi.com/article/10.3390/s21165544/s1, Supplementary Figure S1: Bland–Altman plots of HRV/PRV parameters computed by the Kubios Premium algorithm from 2-min long ECG (indicated as ‘parameter name-ECG’) and PPG (indicated as ‘parameter name-PPG’) recordings captured in healthy individuals under resting conditions; Supplementary Figure S2: Bland–Altman plots of HRV/PRV parameters computed by the Kubios Premium algorithm from 2-min long ECG (indicated as ‘parameter name-ECG’) and PPG (indicated as ‘parameter name-PPG’) recordings obtained from healthy individuals during cold pressor test; Supplementary Figure S3: Bland–Altman plots of HRV/PRV parameters computed by the Kubios Premium algorithm from 2-min long ECG (indicated as ‘parameter name-ECG’) and PPG (indicated as ‘parameter name-PPG’) recordings obtained from diabetic patients under resting conditions; Supplementary Figure S4. Bland–Altman plots of HRV/PRV parameters calculated by the SCN4ALL (indicated as ‘parameter name-SCN4ALL’) and the Kubios Premium HRV (indicated as ‘parameter name-Kubios’) algorithms from 2-min long PPG recordings captured in healthy individuals under resting conditions; Supplementary Figure S5: Bland–Altman plots of HRV/PRV parameters calculated by the SCN4ALL (indicated as ‘parameter name-SCN4ALL’) and the Kubios Premium HRV (indicated as ‘parameter name-Kubios’) algorithms from 2-min long PPG recordings obtained from healthy individuals during cold pressor test; Supplementary Figure S6: Bland–Altman plots of HRV/PRV parameters calculated by the SCN4ALL (indicated as ‘parameter name-SCN4ALL’) and the Kubios Premium HRV (indicated as ‘parameter name-Kubios’) algorithms from 2-min long PPG recordings obtained from diabetic patients under resting conditions.
