# Supplementary material for: Multimodal Assessment of the Pulse Rate Variability Analysis Module of a Photoplethysmography-Based Telemedicine System
Source: Sensors (Basel). 2021 Aug 18;21(16):5544. doi: 10.3390/s21165544 (PMC8401087; doi:10.3390/s21165544)
Supplement: Supplementary file 1 [file sensors-21-05544-s001.zip › sensors-1302188-supplementary.pdf]

Supplementary Material

Supplementary Figure S1: *Bland-Altman plots of HRV/PRV parameters computed by the Kubios Premium algorithm from 2-min long ECG (indicated as 'parameter name-ECG') and PPG (indicated as 'parameter name-PPG') recordings captured in healthy individuals under resting conditions.*

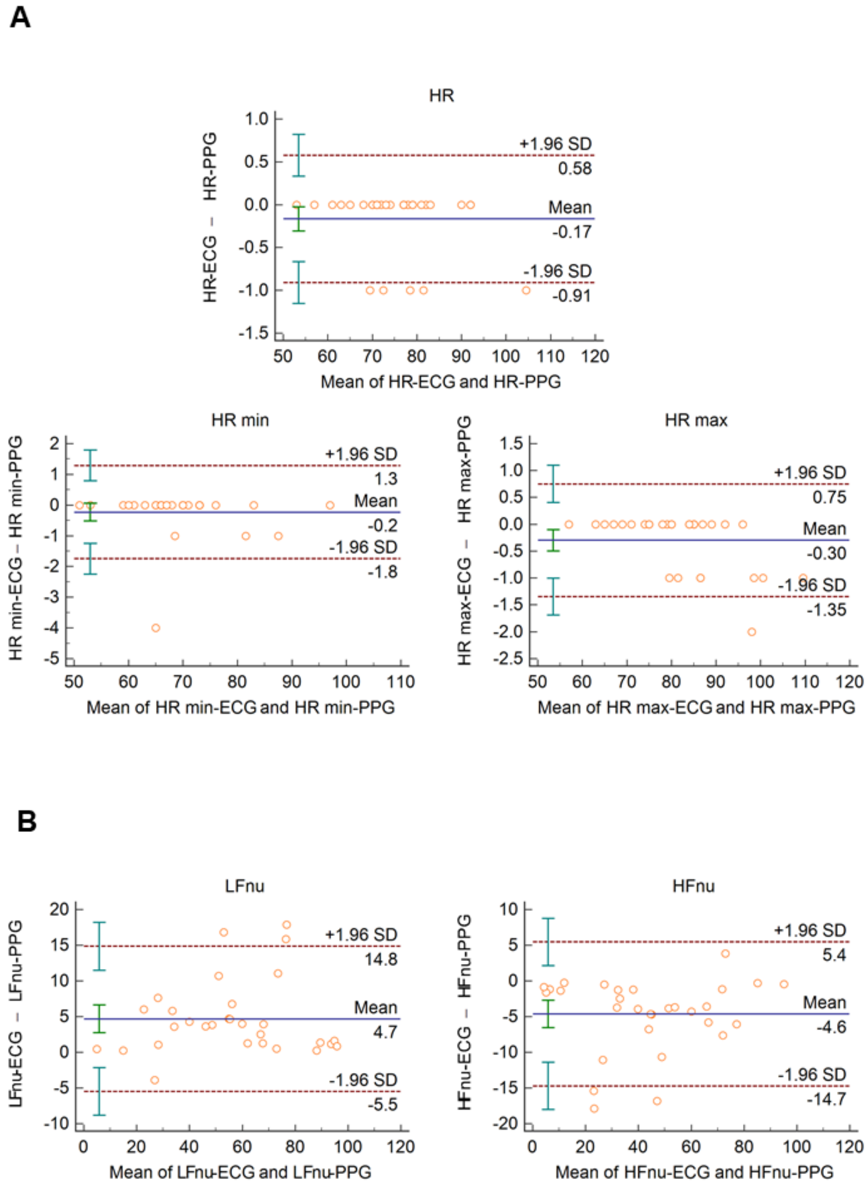

A: *Time-domain parameters: HR (mean heart rate), HR min (minimum heart rate), HR max (maximum heart rate)* ***B:*** *Frequency-domain parameters: LFnu (relative power of the low-frequency band), HFnu (relative power of the high-frequency band). Bias is calculated as the mean of differences (indicated as 'Mean' - blue solid line) and is presented with 95% confidence intervals (green) and +/- 1.96 standard deviations (SD) and their confidence intervals.*

Supplementary Figure S2: *Bland-Altman plots of HRV/PRV parameters computed by the Kubios Premium algorithm from 2-min long ECG (indicated as ‘parameter name-ECG’) and PPG (indicated as ‘parameter name-PPG’) recordings obtained from healthy individuals during cold pressor test.*

A

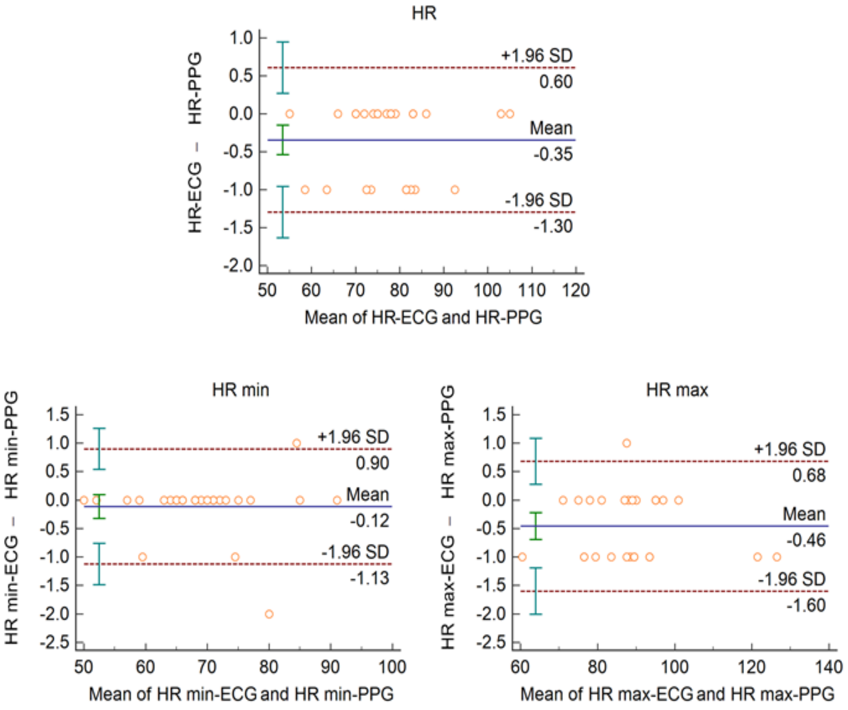

B

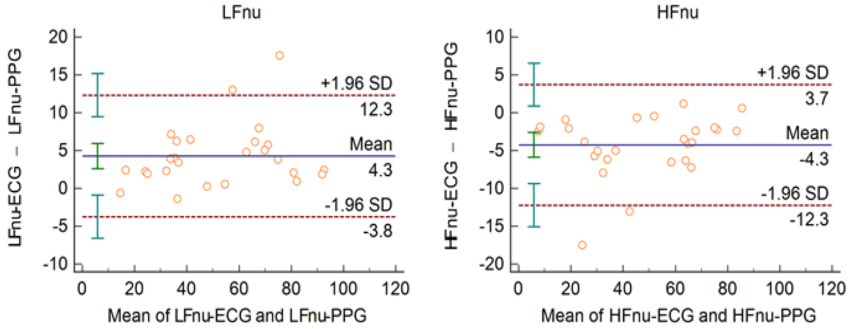

***A**: Time-domain parameters: HR (mean heart rate), HR min (minimum heart rate), HR max (maximum heart rate) **B**: Frequency-domain parameters: LFnu (relative power of the low-frequency band), HFnu (relative power of the high-frequency band). Bias is calculated as the mean of differences (indicated as ‘Mean’ - blue solid line) and is presented with 95% confidence intervals (green) and +/- 1.96 standard deviations (SD) and their confidence intervals.*

.

Supplementary Figure S3: *Bland-Altman plots of HRV/PRV parameters computed by the Kubios Premium algorithm from 2-min long ECG (indicated as 'parameter name-ECG') and PPG (indicated s 'parameter name-PPG') recordings obtained from diabetic patients under resting conditions.*

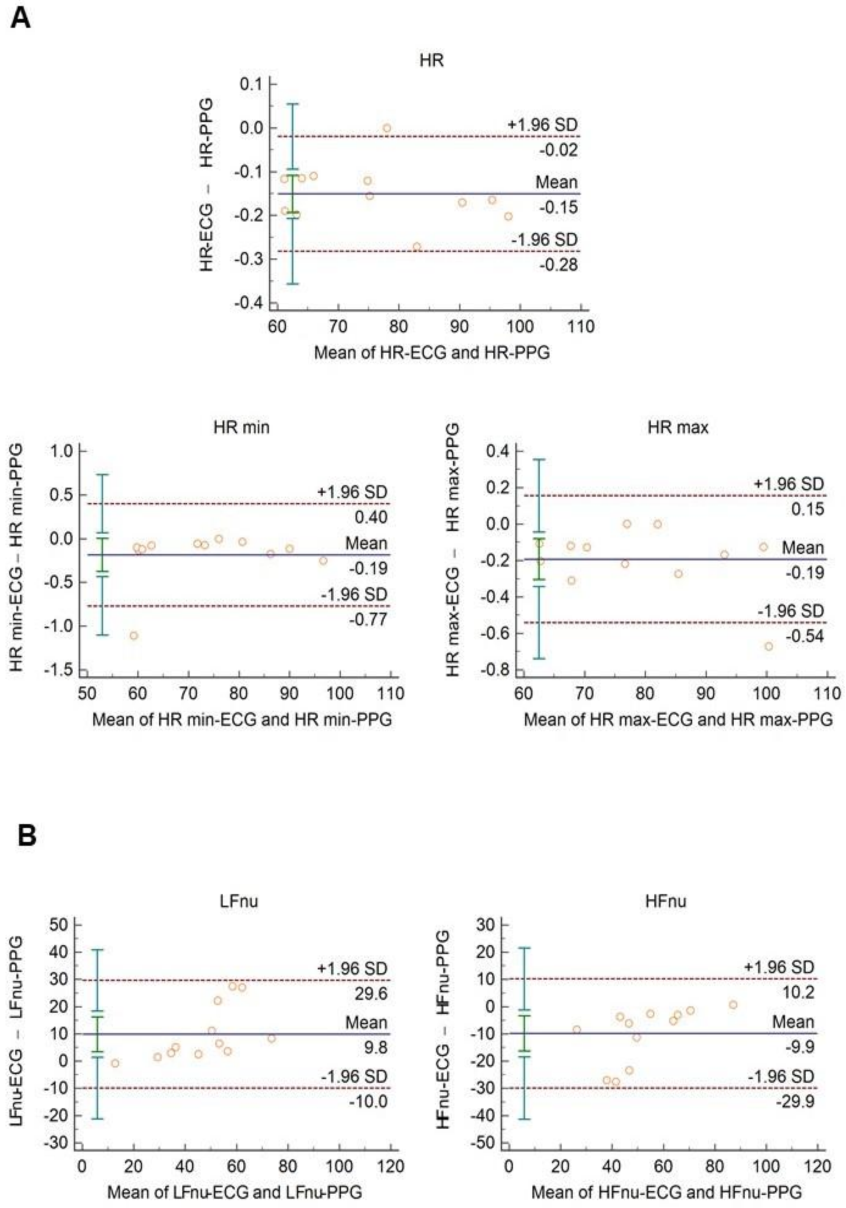

***A**: Time-domain parameters: HR (mean heart rate), HR min (minimum heart rate), HR max (maximum heart rate) **B**: Frequency-domain parameters: LFnu (relative power of the low-frequency band), HFnu (relative power of the high-frequency band). Bias is calculated as the mean of differences (indicated as 'Mean' - blue solid line) and is presented with 95% confidence intervals (green) and +/- 1.96 standard deviations (SD) and their confidence intervals.*

Supplementary Figure S4: *Bland-Altman plots of HRV/PRV parameters calculated by the SCN4ALL (indicated as 'parameter name-SCN4ALL') and the Kubios Premium HRV (indicated as 'parameter name-Kubios') algorithms from 2-min long PPG recordings captured in healthy individuals under resting conditions.*

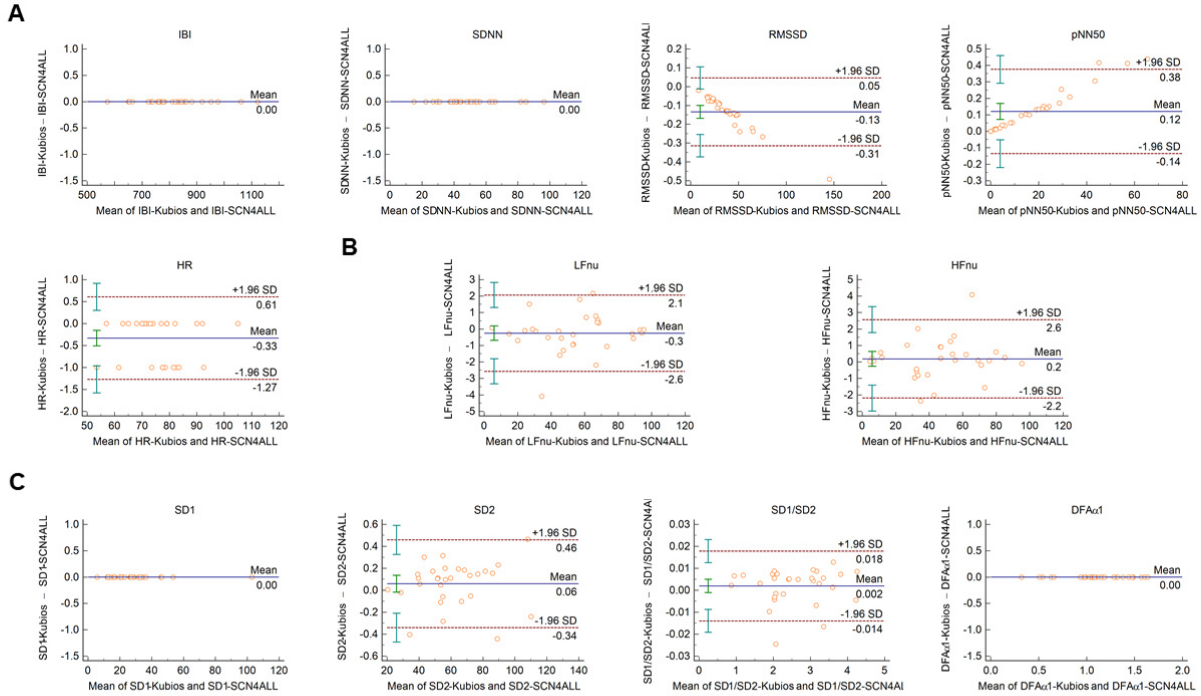

***A**: Time-domain parameters: IBI (interbeat interval), SDNN (the standard deviation of IBIs), RMSSD (the square root of the mean squared differences of successive IBIs), pNN50 (the proportion of differences of successive IBIs exceeding 50 ms), HR (mean heart rate). **B**. Frequency-domain parameters: LFnu (relative power of the low-frequency band), HFnu (relative power of the high-frequency band). **C:** Non-linear parameters: SD1 (Poincaré plot standard deviation perpendicular the line of identity), SD2 (Poincaré plot standard deviation along the line of identity), SD1/SD2 (ratio of SD1-to-SD2), DFAα1 (short term fluctuation slope obtained by detrended fluctuation analysis)*
Bias is calculated as the mean of differences (indicated as 'Mean' - blue solid line) and is presented with 95% confidence intervals (green) and +/- 1.96 standard deviations (SD) and their confidence intervals.

Supplementary Figure S5: *Bland-Altman plots of HRV/PRV parameters calculated by the SCN4ALL (indicated as 'parameter name-SCN4ALL') and the Kubios Premium HRV (indicated as 'parameter name-Kubios') algorithms from 2-min long PPG recordings obtained from healthy individuals during cold pressor test.*

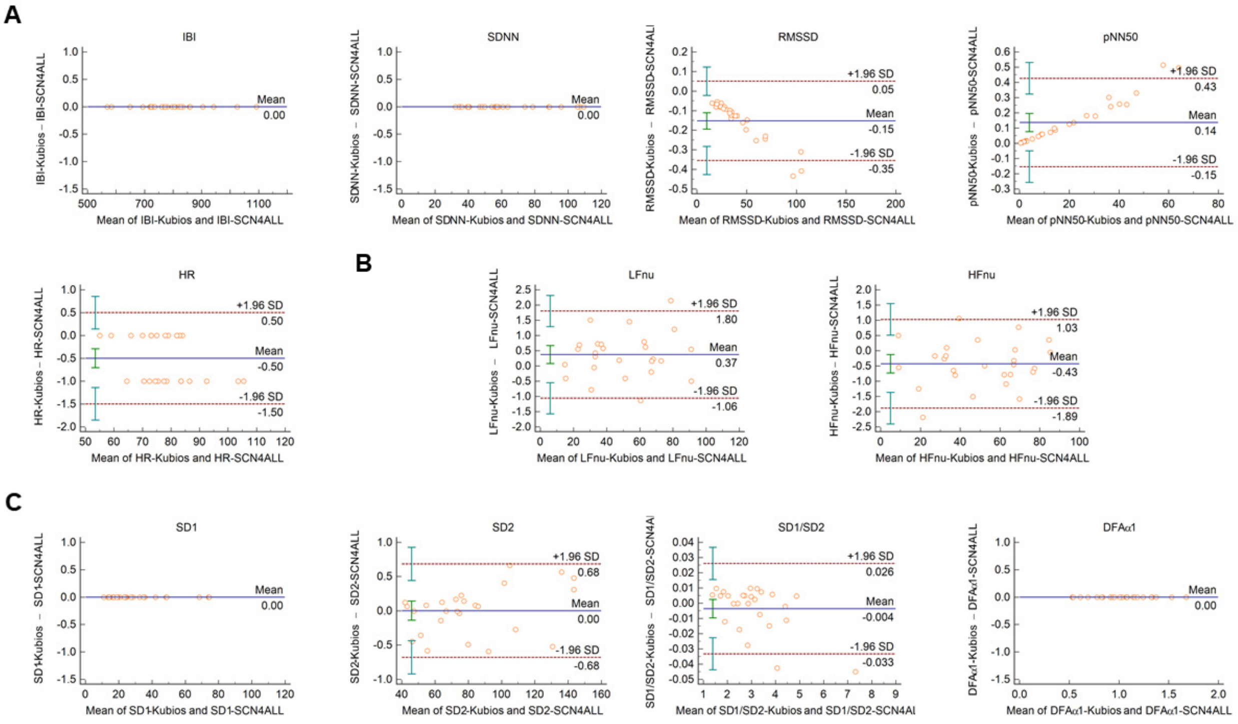

***A**: Time-domain parameters: IBI (interbeat interval), SDNN (the standard deviation of IBIs), RMSSD (the square root of the mean squared differences of successive IBIs), pNN50 (the proportion of differences of successive IBIs exceeding 50 ms), HR (mean heart rate). **B**. Frequency-domain parameters: LFnu (relative power of the low-frequency band), HFnu (relative power of the high-frequency band). **C:** Non-linear parameters: SD1 (Poincaré plot standard deviation perpendicular the line of identity), SD2 (Poincaré plot standard deviation along the line of identity), SD1/SD2 (ratio of SD1-to-SD2), DFAα1 (short term fluctuation slope obtained by detrended fluctuation analysis)*

Bias is calculated as the mean of differences (indicated as 'Mean' - blue solid line) and is presented with 95% confidence intervals (green) and +/- 1.96 standard deviations (SD) and their confidence intervals.

Supplementary Figure S6: *Bland-Altman plots of HRV/PRV parameters calculated by the SCN4ALL (indicated as ‘parameter name-SCN4ALL’) and the Kubios Premium HRV (indicated as ‘parameter name-Kubios’) algorithms from 2-min long PPG recordings obtained from diabetic patients under resting conditions.*

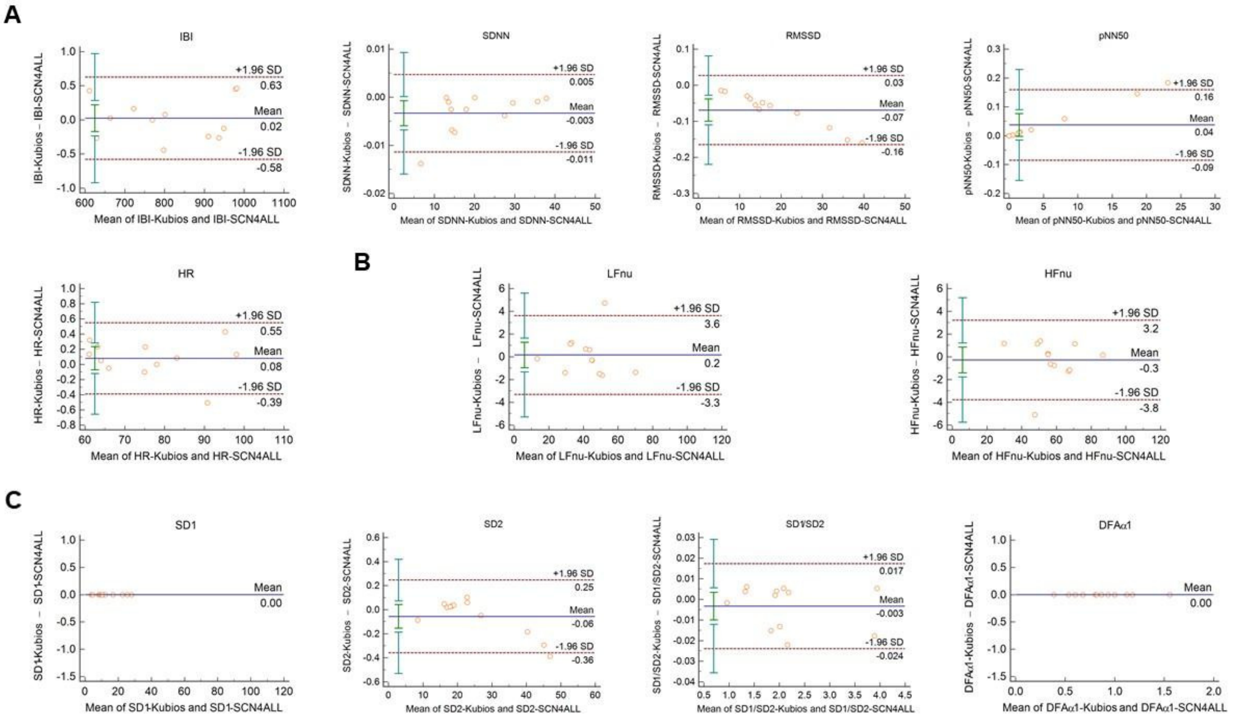

***A**: Time-domain parameters: IBI (interbeat interval), SDNN (the standard deviation of IBIs), RMSSD (the square root of the mean squared differences of successive IBIs), pNN50 (the proportion of differences of successive IBIs exceeding 50 ms), HR (mean heart rate). **B**. Frequency-domain parameters: LFnu (relative power of the low-frequency band), HFnu (relative power of the high-frequency band). **C:** Non-linear parameters: SD1 (Poincaré plot standard deviation perpendicular the line of identity), SD2 (Poincaré plot standard deviation along the line of identity), SD1/SD2 (ratio of SD1-to-SD2), DFAα1 (short term fluctuation slope obtained by detrended fluctuation analysis)*

Bias is calculated as the mean of differences (indicated as ‘Mean’ - blue solid line) and is presented with 95% confidence intervals (green) and +/- 1.96 standard deviations (SD) and their confidence intervals.
